# Hand Knob Stroke Due to an Embolism of a Metastatic Tumor in the Left Atrium

**DOI:** 10.7759/cureus.92332

**Published:** 2025-09-15

**Authors:** Akihiko Mitsutake

**Affiliations:** 1 Department of Neurology, The University of Tokyo, Tokyo, JPN

**Keywords:** cardiac tumors, cortical stroke, stroke, transesophageal echocardiogram, tumor emboli

## Abstract

A stroke confined to the precentral hand knob is uncommon and often mistaken for peripheral neuropathy. Embolic mechanisms are common, most frequently arising from large-artery atherosclerosis, but unusual sources may occur. We describe the case of a 68-year-old man with weakness restricted to the ulnar-sided fingers due to a small infarct in the medial precentral hand knob. Cervical vascular imaging was unremarkable, but echocardiography revealed a highly mobile left atrial mass arising from the ostium of the left upper pulmonary vein. The patient had a history of sublingual gland adenoid cystic carcinoma with metastases to the mediastinum, lungs, and liver. CT confirmed the atrial mass as contiguous with mural thickening, consistent with intracardiac metastasis. Given disseminated disease, supportive care was chosen. To our knowledge, this is the first reported case of a hand knob infarct caused by an embolism from a mobile left atrial metastasis of salivary gland origin, highlighting the need to consider metastatic cardiac tumors, particularly in patients with malignancy and unexplained cortical infarcts.

## Introduction

Hand knob infarction typically presents with isolated hand weakness [1]. Although it accounts for approximately 1% of all ischemic strokes, delays in recognition are common, as it is often mistaken for peripheral neuropathy [2].

The small size of the infarction is the result of occlusion of a distal cortical (M4) branch of the middle cerebral artery. Etiologies are frequently embolic, particularly artery-to-artery embolism from ipsilateral carotid atherosclerosis, but they vary across cohorts. In a review of 150 patients, large-artery atherosclerosis and cardioembolism accounted for 39.3% and 13.3% of cases, respectively, and small-vessel occlusion for 5.3% [3]. By contrast, a single-center series reported small-vessel occlusion and large-artery atherosclerosis as equally common [2]. In another single-center series of 25 cases, supra-aortic atherosclerosis was present in 84%, with artery-to-artery and cardioembolic mechanisms predominating [4].

Primary cardiac tumors are exceptionally rare, with an incidence of approximately 0.02% in autopsy series [5]. Cardiac myxoma is the most common type, representing roughly half of all primary cardiac neoplasms and up to two-thirds of benign lesions, and carries a well-recognized risk of embolization and stroke [6]. In contrast, metastatic cardiac involvement is considerably more frequent, as autopsy studies have reported incidences of 2.3-18.3% among patients with known malignancies [7]. The most common primaries are lung, breast, and hematologic cancers [7,8].

We report the case of an isolated hand knob infarct caused by a mobile left atrial metastasis from sublingual gland carcinoma, underscoring the need to consider intracardiac metastatic sources when cervical vascular imaging is unrevealing.

## Case presentation

**Figure 1:**
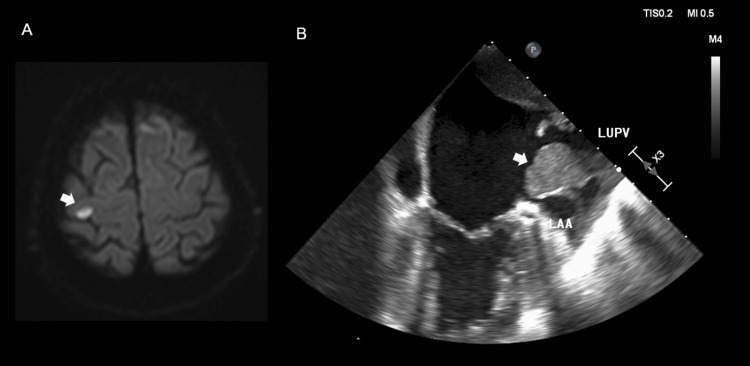
Brain MRI and transesophageal echocardiography. (A) Brain MRI shows hyperintensity in the right precentral knob on diffusion-weighted imaging (arrow) with decreased apparent diffusion coefficient (not shown). (B) Transesophageal echocardiography shows tumor invasion into the left atrium and a tumor mass with mobility (8 × 6 mm, arrow) at the left pulmonary vein ostium. The arrow indicates the mass. LUPV: left upper pulmonary vein; LAA: left atrial appendage

A right-handed 68-year-old male smoker with no other vascular risk factors suddenly noticed difficulty moving the fourth and fifth digits of his left hand while attempting to fold a compact umbrella. The symptoms had improved by the time he presented to a clinic later the same day, and he was referred to our neurology clinic for further evaluation the following day. Neurological examination performed two days after symptom onset revealed mild weakness in the fourth and fifth digits of the left hand without sensory loss. Weakness involved the interossei and the digital flexors/extensors, but there was no clinically evident weakness of the wrist or other forearm muscles. Routine laboratory tests and electrocardiography were unremarkable. Brain MRI demonstrated a punctate hyperintense lesion on diffusion-weighted imaging in the medial portion of the right precentral gyrus, corresponding to the cortical territory governing the ulnar-sided fingers (Figure 1A). Magnetic resonance angiography of the brain and neck and carotid ultrasonography showed no evidence of intra- or extracranial arterial stenosis. Transthoracic echocardiography demonstrated an echogenic mass in the left atrium, and transesophageal echocardiography revealed an 8 × 6 mm pedunculated, highly mobile tumor arising from the ostium of the left upper pulmonary vein and intermittently prolapsing into the atrial cavity (Figure 1B).

**Figure 2:**
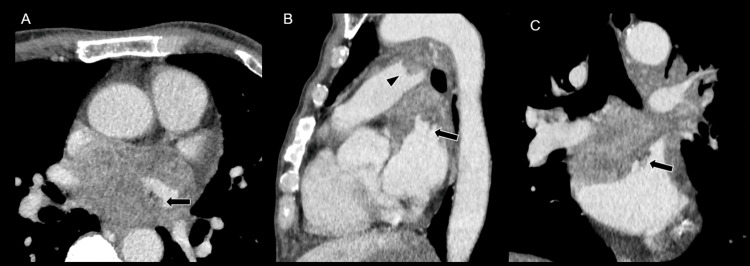
Chest CT. Chest contrast-enhanced CT demonstrates left atrial invasion (A-C, arrows), and pulmonary artery invasion (B, arrowhead) of the mediastinal metastatic tumor.

Contrast‑enhanced chest and abdominal CT showed multiple pulmonary and hepatic nodules, along with mural thickening of the left atrial roof contiguous with the mass (Figures 2A-2C).

The patient had stage III sublingual gland adenoid cystic carcinoma, which was treated surgically 13 years earlier. He subsequently developed metastases to the mediastinum, lungs, and liver. His clinical course remained indolent, and, in the absence of effective chemotherapy, he was managed conservatively.

A diagnosis of recurrent metastatic disease with presumed tumor embolism was made based on the combination of acute cerebral infarction, a left atrial tumor, and disseminated metastases. Given the disseminated nature of the cancer, surgical excision or anticoagulation was considered unlikely to improve prognosis. The patient opted for supportive care and experienced no further neurological events during a three-month follow-up period.

## Discussion

In the present case, the infarct selectively involved the medial portion of the precentral knob, consistent with weakness restricted to the ulnar-sided fingers [9]. The absence of carotid stenosis on vascular imaging, together with the identification of a mobile left atrial mass on echocardiography, indicated a cardiac embolic source rather than artery-to-artery embolism [2-4].

Previous studies have shown that embolic mechanisms are common in hand knob infarctions. Most reported cases were attributed to large-artery atherosclerosis [3,4]. In some cohorts, however, small-vessel occlusion was found to be equally frequent [2]. Our patient’s stroke pattern could also be classified as small-vessel occlusion, but the underlying mechanism was fundamentally different from intrinsic small-vessel disease, such as lipohyalinosis or arteriolosclerosis. In this case, the infarction was caused by tumor emboli originating from a metastatic cardiac tumor, representing an unusual embolic mechanism. Cardiac tumors are established sources of emboli, particularly cardiac myxomas [10,11]. The most common primaries are lung, breast, and hematologic malignancies [7,8], whereas salivary gland carcinomas, especially those of the sublingual gland, rarely metastasize to the heart [12]. To our knowledge, no prior report has documented an isolated hand knob infarct caused by embolism from a mobile left atrial metastasis of sublingual gland carcinoma, thereby broadening the spectrum of cancer-related stroke mechanisms.

This case underscores a common diagnostic pitfall in isolated hand weakness: subtle cortical strokes can mimic peripheral neuropathies and lead to diagnostic delay [2]. In patients with a history of cancer who present with isolated hand weakness, early MRI is warranted even when cortical signs are subtle. If vascular imaging of the brain and neck is unrevealing, a systematic evaluation for intracardiac sources, including metastatic lesions, should follow. Notably, high mobility of intracardiac masses has been linked to increased embolic potential in myxoma cohorts [10,11]. This principle likely extends to other intracardiac tumors [8,13], including metastatic lesions, such as in the present case.

Regarding the management of metastatic cardiac tumors, the role of surgery remains debated. Selected patients with isolated cardiac metastasis and preserved performance status may benefit from resection [8]. In the present case, widespread metastatic involvement and patient preference informed a palliative approach, which was consistent with these recommendations.

## Conclusions

This case broadens the recognized etiologic spectrum of isolated hand knob infarction to include embolism from metastatic cardiac tumors of salivary gland origin. In patients with malignancy who present with isolated hand weakness, a prompt brain MRI is warranted to identify cortical infarcts. When vascular imaging of the brain and neck is non-revealing, cardiac evaluation should follow. Although cardiac evaluation is routinely performed in the diagnostic workup of ischemic stroke, our case highlights that it should not be overlooked even in seemingly minor cortical infarctions such as hand knob strokes, where uncommon embolic sources such as metastatic cardiac tumors may be identified. Mobile intracardiac masses are highly prone to causing embolism, and this risk appears equally relevant for metastatic lesions. Management should be guided by oncologic history, lesion mobility, and the overall metastatic burden.
